# Effect of tibialis anterior muscle resistance training on ankle and foot dorsum extension function in hypertensive cerebral hemorrhage hemiplegia patients: A randomized controlled trial

**DOI:** 10.1097/MD.0000000000033827

**Published:** 2023-08-04

**Authors:** Qiaoliang Li, LiGang Chen

**Affiliations:** a Affiliated Hospital of Southwest Medical University, Luzhou, China.; b Department of Neurosurgery, The Affiliated Hospital of Southwest Medical University.

**Keywords:** cerebral hemorrhage, foot back stretch, hemiplegia, hypertension, intensive training, pretibial muscle, resistance training, triceps calf

## Abstract

This study aimed to investigate the effect of tibialis anterior muscle resistance training on ankle and foot dorsum extension function in patients with hemiplegia caused by hypertensive cerebral hemorrhage.

**Methods:**

Fifty cases of hypertensive cerebral hemorrhage in patients with hemiplegia were selected according to the random number table method. The patients were divided into the treatment group and control group. Each group included 25 cases, and the treatment group was given routine rehabilitation treatment and passive and active foot back stretch training (300 times/d). The control group received conventional rehabilitation treatment. The conventional rehabilitation treatment included stretching, muscle strengthening and other conventional rehabilitation treatment techniques. Surface electromyography was used to evaluate the muscle strength and tension of the triceps and tibialis anterior muscles of the affected side of the patients before and after treatment. The root mean square value of the surface electromyography (RMS) of the passive triceps extension before and after treatment was used to evaluate the muscle strength and tension of the affected side. The ratio of passive traction and relaxation of the triceps before and after treatment and the ratio of active contraction and relaxation of the tibialis anterior muscle before and after treatment were recorded.

**Results:**

There was no significant difference in surface electromyography between the 2 groups before treatment (*P* > .05). After 2 months of treatment, the results of patients in both groups improved compared with those before treatment. The RMS of triceps in the treatment group was significantly lower than that in the control group, and the ratio of RMS of triceps in the treatment group was significantly lower than that in the control group. The RMS during active contraction and the RMS ratio between active contraction and relaxation of the tibialis anterior muscle in the treatment group were significantly higher than those in the control group (*P* < .05).

**Conclusions:**

Tibialis anterior muscle resistance training can effectively improve the strength of the tibialis anterior muscle in patients with hemiplegia caused by hypertensive cerebral hemorrhage, reduce tension in the triceps calf muscle, and improve ankle joint function and foot dorsum extension.

## 1. Introduction

Stroke is a common and frequently occurring condition in clinical practice. The incidence of cerebral hemorrhage in all stroke subtypes is second only to that of ischemic stroke. According to a domestic epidemiological survey, the proportion of cerebral hemorrhage in China is higher than that in Western countries, accounting for 18.8% to 47.6% of strokes,^[1,2]^ half of which are caused by hypertension.^[3]^ The basal ganglia are a common site of cerebral hemorrhage,^[4]^ and limb paralysis often occurs after damage to this region.^[5]^ The limb muscles on the affected side and extensor muscle spasms are the main muscles in the lower limb, including the quadriceps femoris, triceps calf. Hemiplegic patients have high muscle tension of the adductor muscle and triceps muscle of the lower extremity, hip abduction disorder, ankle and foot dorsum extension disorder and other clinical signs.^[6–8]^ Approximately 80% of patients cannot recover their self-care ability after 6 months, which places a heavy burden on society and families.^[1]^

Improving ankle and foot dorsum extension function and the gait of patients with foot drop,^[9]^ and reducing triceps muscle tension ankle dysplasia, varus, and other complications^[10,11]^ have become key issues in the treatment of hemiplegia. The search for effective treatment options has become the clinical consensus.

Currently, the main treatments for hemiplegia are exercise therapy, forced exercise therapy, acupuncture, and physiotherapy.^[12,13]^ Exercise therapy is one of the most important treatments. Intensive exercise therapy has a good effect on long-term prognosis after stroke.^[14]^ However, there is still a lack of effective and low-cost foot dorsiflexion exercise therapy, and most patients require long-term treatment in medical institutions. Patients find it difficult to maintain treatment because of the efficacy, cost and other reasons. Therefore, it is of great clinical, economic and social significance to explore an economic, efficient and convenient treatment method to reduce the triceps muscle tension of the calf, improve the muscle strength of the tibialis anterior muscle of the calf, and improve the dorsum extension function of the affected side of the lower limb. This study combined tibialis anterior muscle resistance training for the treatment of ankle joint and foot dorsiflexion dysfunction in hypertensive cerebral hemorrhage hemiplegia patients, and a surface electromyography (SEMG) measuring instrument^[15,16]^ was used to observe its effects on ankle dorsiflexion function, tibialis anterior muscle strength, and triceps muscle tension on the affected side of hemiplegic patients, which are reported as follows.

## 2. Clinical data

### 2.1. Case selection criteria

In accordance with the diagnosis of hypertensive intracerebral hemorrhage, the diagnostic criteria refer to the diagnostic criteria of the Chinese Multidisciplinary Diagnosis and Treatment Guidelines for hypertensive intracerebral hemorrhage^[17]^: Hypertensive intracerebral hemorrhage refers to sudden cerebral parenchymal hemorrhage in the basal ganglia, thalamus, ventricle, cerebellum and brainstem of a patient with a clear history of hypertensive venereal disease.

Inclusion criteria were as follows: patients aged 30 to 70 years, patients who met the diagnostic criteria for hypertensive intracerebral hemorrhage and were diagnosed with unilateral involvement on computed tomography or magnetic resonance imaging, the disease course was >6 months, vital signs were stable, neurological symptoms were no longer progressive, consciousness was clear, and the patient could cooperate with the command movement, and the muscle strength of the tibial anterior muscle decreased, and the muscle strength was evaluated as grades 1 to 5 according to the free-hand manual muscle testing grading method. All patients and their family members agreed to participate in the study and voluntarily signed informed consent forms.

The exclusion criteria were as follows: patients with unstable vital signs or severe complications (cerebral hernia, myocardial infarction, severe liver and kidney dysfunction, severe infection, or venous thrombosis of both lower extremities), patients with intracerebral hemorrhage caused by trauma, coagulopathy, hematological diseases, or neoplastic diseases were excluded. Patients with consciousness disorders, sensory aphasia, etc., patients with other conditions of lower limb dysfunction (spinal cord injury, amputation, severe bone and joint diseases, etc.), and patients with conditions that cause balance dysfunction (vertigo, vestibular dysfunction, cerebellar lesions).

### 2.2. General data

A total of 50 patients with craniocerebral injury were admitted to the Department of Neurosurgery, and the sample size was determined by referring to previous similar randomized controlled experiments. Patients from the Second People’s Hospital of Yibin from January 2020 to January 2022 were selected and divided into treatment and control groups according to the random number table method with 25 cases in each group. This study was approved by the ethics committee of the Second People’s Hospital of Yibin. There were no significant differences in sex, age, course of disease, blood loss, muscle strength, or muscle tension between the 2 groups (*P* > .05), which were comparable (Table 1).

**Table 1:** Comparison of general data between 2 groups (*X* ± *s*).

	The number of	Gender	Age	Course of the disease	The bleeding	Muscle tension	Strength
The treatment group	25	Male 23/female 2	57.33 ± 10.03	18.5 ± 11.38	28.06 ± 18.45	2.0 ± 1.19	2.57 ± 1.27
The control group	25	Female 24/male 1	48.83 ± 11.86	17 ± 13	20.6 ± 18.85	2.14 ± 1.21	2.28 ± 1.88

## 3. Method

### 3.1. Treatment methods

Methods rehabilitation training was carried out by the same group of rehabilitation therapists.

The control group was given conventional rehabilitation techniques such as stretching, muscle strength, balance and wearing orthoses. According to the actual movement function in patients with grade wearing knee ankle foot orthoses or ankle foot orthoses, training content included physical therapy (passive training, sit-ups, bridge type double lower limb joint movement, roll over training, seat, aided by sitting and standing balance training, orthoses wear to take off the train, aided by up and down the stairs and training), trunk core muscle group training, acupuncture and moxibustion treatment, cramps machine, etc. The training was performed once a day for 30 minutes each time for 8 weeks.

The treatment group included passive and active dorsalis pedis training 300 times/d and was divided into 3 to 5 groups according to the degree of tolerance of children and their families. Each group had 60 to 100 points to complete 3 to 5 times a day, with a routine rehabilitation training and training cycle for 8 weeks. Muscle strength was evaluated as grades 1 to 5 according to the free-hand manual muscle testing grading method. Grades 1 and 2 were trained with active exercise plus a lot of auxiliary exercise; grades 3 and 4 were trained with active exercise plus a little resistance exercise; and grade 5 was trained with active exercise plus a lot of resistance exercise.

### 3.2. Observation indices

Before treatment and 2 months after treatment, the electromyography (EMG) of the affected side of the tibialis anterior and peroneus longus muscles was recorded using the SA7550 surface EMG analysis system produced by Nanjing Weis Medical Co., Ltd. Before and after treatment, the root mean square (RMS) value of the triceps leg passive extension,^[18,19]^ ratio of the triceps leg passive extension and relaxation,^[20,21]^ ratio of the active contraction of the tibialis anterior muscle before and after treatment, and ratio of the active contraction and relaxation of the tibialis anterior muscle before and after treatment were evaluated. The formula of RMS was as follows: RMS ratio (%) =RMS measured muscle (passive traction or active contraction)/RMS measured muscle (relaxation) multiplied by 100%.

During the evaluation, the patient rested quietly for 5 minutes, the room temperature was approximately 30 to 35 °C, the lower limbs were placed in an upright sitting position, the lower legs were exposed, the skin was wiped with alcohol, the triceps electromyography electrode was connected, and the tibialis anterior and triceps muscles were fully extended. The tibialis anterior and triceps muscles are channels A and B, respectively.

The muscles of channel A were actively contracted in the following steps: contract, relax, contract, relax, contract, and relax.

The channel B muscles passive draft were the following: pull, relax, pull, relax, pull, and relax

The SEMG signal was collected, and the system automatically analyzed the root-mean-square value of the surface EMG signal.

### 3.3. Statistical analysis

SPSS17.0 software (International Business Machine, Armonk, NY) was used to analyze SPSS data. The data did not conform to a normal distribution; therefore, the nonparametric rank sum test (Mann–Whitney *U* test) was used to analyze the difference between the data of each group before and after treatment; *P* < .05, as shown in Table 2, was considered statistically significant.

**Table 2:** IQR comparison of treatment data between the 2 groups (IQR).

	The treatment group			The control group			*P* value
	Before the treatment	After treatment	Difference	Before the treatment	After treatment	Difference	
Passive traction of the calf triceps	36.77 (18.28–102.27)	14.25 (9.00–39.47)	9.33 (88.44–7.47)	61.26 (32.97–89.64)	39.71 (37.14–90.59)	4.17 (27.3–6.64)	.002
Ratio of passive extension to passive relaxation of calf triceps	8.56 (7.46–13.76)	5.47 (3.83–6.24)	3.05 (5.99–1.09)	6.20 (4.11–9.00)	5.17 (4.39–6.82)	0.07 (2.57–0.68)	.001
Active contraction of tibialis anterior muscle	38.15 (14.88–122.49)	67.15 (35.17–144.69)	24.17 (9.40–28.7)	19.06 (16.72–149.60)	38.26 (19.28–154.88)	5.24 (0.40–16.09)	.001
Ratio of active contraction to active relaxation of tibialis anterior muscle	8.48 (3.56–21.33)	18.81 (5.13–54.02)	13.79 (2.53–33.7)	9.80 (6.01–13.10)	13.44 (6.67–17.39)	2.19 (1.1–33.7)	.002

IQR = interquartile range.

## 4. Results

### 4.1. Comparison of patients before and after training

Twenty-five patients were enrolled in the experimental group, and 24 patients were enrolled in the control group. One patient in the control group had another sudden cerebral hemorrhage after enrollment, so he dropped out halfway. In the treatment group, the RMS difference in triceps crus passive extension before and after treatment and the ratio difference in RMS between passive extension and relaxation of the triceps crus were significantly lower than those in the control group (*P* < .05). Before and after treatment, pretibial muscle active contraction of the RMS difference, pretibial muscle contraction and relaxation activity when the ratio of the RMS difference was significantly higher than that of the control group was better than that before training (*P* < .05) (see Table 1). The data show that 2 groups of leg triceps muscle tension and pretibial muscle strength improved significantly earlier, but the treatment group improved better than the control group.

Resistance training can improve the strength of the tibialis anterior muscle, reduce the tension of the triceps calf muscle, and improve ankle and foot dorsum extension function in patients with hemiplegia caused by hypertensive cerebral hemorrhage. In addition, this exercise is easy for patients to learn and master and can reduce medical expenses.

## 5. Discussion

### 5.1. Passive traction SEMG data of the triceps crus in both groups are presented in the third row of Table 2

Compared with the observation group, the SEMG of the treatment group was higher.

The treatment group was 9.33 (88.44–7.47) and the control group was 4.17 (27.3–6.64).

The difference between the 2 groups was statistically significant (*P* = .002).

The surface electromyography during passive traction of the triceps crus indicated triceps crus muscle tension. The above data showed that the triceps calf muscle tension of the treatment group was lower than that of the observation group, and the difference was statistically significant.

Ratio of passive extension to passive relaxation SEMG data of the triceps crus in the 2 groups are presented in Row 4 of Table 2.

Compared with the observation group, the value of the treatment group was higher.

The treatment group 3.05 (5.99–1.09).

The control group 0.07 (2.57–0.68).

The difference between the 2 groups was statistically significant (*P* = .001).

To reduce the measurement error, the triceps calf muscle tension in the treatment group was lower than that in the observation group, and the above data showed that the triceps calf muscle tension in the treatment group was lower than that in the observation group, and the difference was statistically significant.

Row 5 of Table 2 shows the active contraction surface EMG data of the tibialis anterior muscle in both groups.

Compared with the observation group, the EMG of the treatment group was higher.

The treatment group was 24.17 (9.40–28.7) and the control group was 5.24 (0.40–16.09).

The difference between the 2 groups was statistically significant (*P* = .001).

The surface electromyography during the active contraction of the anterior tibial muscle suggested the strength of the anterior tibial muscle. The above data showed that the strength of the anterior tibial muscle in the treatment group was higher than that in the observation group, and the difference was statistically significant.

Row 6 of Table 2 shows the ratio of the surface EMG data of active contraction to active relaxation of the tibialis anterior muscle for both groups.

Compared with the observation group, the value of the treatment group was higher.

The treatment group was 13.79 (2.53–33.7) and the control group was 2.19 (1.1–33.7).

The difference between the 2 groups was statistically significant (*P* = .002).

The comparison of the data obtained during active contraction of the tibialis anterior muscle with the data obtained during relaxation was performed to reduce measurement error. The above data showed that the muscle strength of the tibialis anterior muscle in the experimental group was higher than that in the observation group during active contraction, and the difference was statistically significant.

Hemiplegia patients’ high leg triceps muscle tension is one of the main factors that limits walking.^[22]^ These study results show that pretibial muscle resistance training, compared with conventional rehabilitation training, improves the pretibial muscle strength, reduces the crus of the triceps muscle tension, and improves the ankle foot flexion and extension function in patients with hemiplegia spasm.

This is related to the high frequency of intensive training. The training frequency of conventional rehabilitation training for the tibialis anterior muscle is often less than 100 times per day, while the training frequency designed in this study is 300 times per day, which is several times higher than that of conventional rehabilitation training, and the high frequency of intensive training can improve patients’ limb control.^[23,24]^

This study is similar to the article by Yoo DY from Korea “Impact of intensive rehabilitation on long-term prognosis after stroke: A Korean nationwide retrospective cohort study,” which shows that intensive rehabilitation has long-term positive effects on stroke patients.^[14]^ The conclusion of this paper is similar, but the content is more specific.

The intensive training method designed in this study is simple and easy to learn, and patients or their family members can master it proficiently after training. Patients can train independently at any time, with strong active participation. However, routine rehabilitation training involves many complex movements, in which patients and their family members have a limited grasp of the training, and active participation is weak. The rehabilitation effect of patients with good active participation is better than that of patients with weak active participation.^[25]^

Hemiplegia requires long-term treatment, and many patients cannot be treated in medical institutions for a long time. After mastering this intensive training method, patients and their family members can train at home by themselves, which can reduce medical expenses and the risk of poverty caused by illness.

However, intensive training of the tibialis anterior muscle requires a high degree of cooperation among the patients, therapists and their families. During training, concentration should be on the muscle, movement should be slow, patients’ tolerance should be taken as appropriate, and overtraining should not aggravate the injury of patients.

### 5.2. Limitations of the study

This study had the limitation of a small sample size of stroke patients from a single medical center, which does not provide a population-based representation of stroke patients. Additionally, we did not observe the recovery of foot dorsiflexion in patients with longer workouts. In the future, larger multicenter studies could be performed. The long-term efficacy of the treatment and the function of foot dorsiflexion in hemiplegic patients were observed.

## 6. Conclusions

In conclusion, based on routine rehabilitation training, tibialis anterior muscle resistance training for hemiplegic patients can improve walking ability, reduce treatment costs, and facilitate home rehabilitation training, which is worthy of clinical promotion and application.

This study has some limitations. For example, the sample size included in this study was small, which was not sufficient to represent the situation of all patients. Larger sample sizes and more in-depth research are expected in the future.

## Author contributions

**Conceptualization:** Qiaoliang Li.

**Data curation:** Qiaoliang Li.

**Formal analysis:** Qiaoliang Li.

**Funding acquisition:** Qiaoliang Li.

**Investigation:** Qiaoliang Li.

**Methodology:** Qiaoliang Li.

**Project administration:** LiGang Chen.

**Resources:** Qiaoliang Li.

**Software:** Qiaoliang Li.

**Supervision:** LiGang Chen.

**Validation:** Qiaoliang Li.

**Visualization:** Qiaoliang Li.

**Writing – original draft:** Qiaoliang Li.

**Writing – review & editing:** Qiaoliang Li.
